# Voltage-Dependent Anion Channel 2 of *Arabidopsis thaliana* (AtVDAC2) Is Involved in ABA-Mediated Early Seedling Development

**DOI:** 10.3390/ijms10062476

**Published:** 2009-05-26

**Authors:** Jinping Yan, Han He, Shibo Tong, Wanrong Zhang, Jianmei Wang, Xufeng Li, Yi Yang

**Affiliations:** 1 Key Laboratory of Bio-resources and Eco-environment of the Ministry of Education, College of Life Science, Sichuan University, Chengdu 610064, China; E-Mails: jpyan2007@gmail.com (J.Y.); hanh1204@yahoo.com.cn (H.H.); tsb821@163.com(S.T.); gladys9835@yahoo.com.cn (W.Z.); wangjianmei06@gmail.com (J.W.); lixufeng0507@gmail.com (X.L.); 2 Biotechnology & Genetic Germplasm Institute, Yunnan Academy of Agricultural Sciences, 9# Xueyun Road, Kunming 650223, China

**Keywords:** Arabidopsis thaliana, voltage-dependent anion channel, abscisic acid, ABA signaling

## Abstract

The voltage-dependent anion channel (VDAC) is the major transport protein in the outer membrane of mitochondria and plays crucial roles in energy metabolism, apoptosis, and metabolites transport. In plants, the expression of VDACs can be affected by different stresses, including drought, salinity and pathogen defense. In this study, we investigated the expression pattern of AtVDAC2 in *A. thaliana* and found ABA suppressed the accumulation of AtVDAC2 transcripts. Further, phenotype analysis of this VDAC deregulated-expression transgenic Arabidopsis plants indicated that AtVDAC2 anti-sense line showed an ABA-insensitivity phenotype during the early seedling development under ABA treatment. The results suggested that AtVDAC2 might be involved in ABA signaling in *A. thaliana*.

## 1. Introduction

The voltage-dependent anion channel (VDAC) is the major channel on the outer membrane of mitochondria. VDAC mediates the exchange of many metabolites such as ions, ATP, and ADP between the cytosol and mitochondria [1,2]. VDAC is also a major component of the tRNA import machinery in plant mitochondria [3]. Therefore, the state of this channel (opening or closure) would affect the normal functions of the mitochondria, the cells and even the individual.

Earlier studies on VDAC mainly focused on its isoforms in animals and yeasts [4,5]. In animal cells, VDAC plays an important role in apoptosis by participating in the release of cytochrome C [6]. In yeast, studies using *Saccharomyces cerevisiae* mutants depleted of either isoform of VDAC, showed that both the cytosol and mitochondria redox states depend on the presence of VDAC [7]. On the basis of these earlier studies, Lemasters and Holmuhamedov considered VDAC as a “governator” of mitochondrial activity and function [8].

However, the researches on plants mainly focus on the identification and the expression pattern analysis of the VDAC isoforms. Up to now, VDAC isoforms have been identified from maize, rice [1,9], wheat [10], rape [11], tobacco [12], and Arabidopsis [13]. The expression pattern analysis revealed that VDAC affected plant response to different stresses, including drought, heat shock, salinity [11,14], as well as defense against pathogen [12].

Abscisic acid (ABA), as an endogenous phytohormone, is involved in plant response to abiotic stresses imposed by salt, cold, drought and wounding, or biotic abiotic stress by pathogen [15,16]. Until recently, there is a lack of knowledge about the relationship between these two important elements, VDAC and ABA.

Using the yeast two-hybrid system, our earlier studies have revealed that one isoform of AtVDACs, AtVDAC2 (At5g67500), is a potential protein interaction partner of one ABA signal component, which is also an interaction partner of ABI1 and ABI2. In this paper, we sought to investigate whether AtVDAC2 involved in the response to ABA in plant. Using RT-PCR and the protoplast transient expression system, the analysis on the expression pattern of AtVDAC2 under ABA treatment showed that ABA suppressed the accumulation of AtVDAC2 transcripts. And further phenotype analysis of the stable AtVDAC2 transgenic plants confirmed that AtVDAC2 involved in ABA signaling.

## 2. Results and Discussion

### 2.1. ABA Suppressed the Accumulation of AtVDAC2 Transcripts

**Figure 1.**
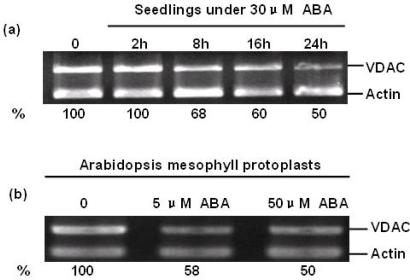
Effect of ABA on AtVDAC2 gene expression at the transcriptional level detected by semi-quantitative PCR. (a) The effect of ABA on AtVDAC2 mRNA level. Four-week old Arabidopsis seedlings were treated with 30μM ABA for 0, 2 h, 8 h, 16 h and 24 h, respectively. (b) The relative AtVDAC2 abundance in Arabidopsis mesophyll protoplasts under 5 μM, 50 μM ABA treatment. The quantitative analysis of the PCR signal performed with imaging software (Gel-Pro analyzer 3.0) and the bands intensities relative to their actin.

ABA regulates the expression levels of a range of genes including those involved in both ABA metabolism and signaling [17,18]. To investigate whether ABA can change the expression of AtVDAC2 at the transcriptional level, firstly, four-week old Arabidopsis seedlings were treated with 30μM ABA for 0, 2 h, 8 h, 16 h and 24 h. Then, the relative AtVDAC2 abundance was detected by semi-quantitative RT-PCR. The result indicated that ABA could suppress the expression of AtVDAC2 to about 100%, 68%, 60% and 50% of the control after 2 h, 8 h, 16 h and 24 h treatment by 30 μM ABA, respectively (Figure 1a).

As a versatile cell system for transient gene expression analysis, the relative AtVDAC2 abundance in Arabidopsis mesophyll protoplasts under ABA treatment was investigated. Arabidopsis mesophyll protoplasts were isolated from three or four-week old seedlings and treated with ABA (5 μM, 50 μM) overnight. Coinciding with the result of seedlings, ABA could suppress the expression of AtVDAC2 in Arabidopsis mesophyll protoplasts with an approximately 50% reduction of wild type. However, there was no significant difference in the accumulation of AtVDAC2 transcripts between the treatments with 5 μM and 50 μM ABA (Figure 1b).

### 2.2. Regulation of AtVDAC2 Promoter by ABA in the Protoplast Expression System

**Figure 2.**
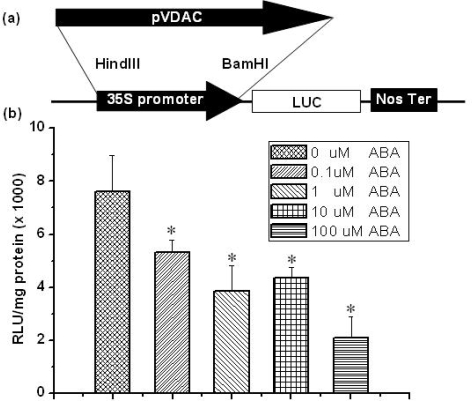
The relative activity of 5′ upstream region of AtVDAC2 regulated by ABA. (a) Construction of the pBI221-pVDAC::LUC vector for the transient gene expression in Arabidopsis mesophyll protoplasts. (b) The luciferase activity of AtVDAC2 promoter in protoplasts was regulated by different level of ABA (0.1, 1, 10 and 100 μM). Luciferase activity was means ± SD (n = 3) from one of three independent experiments. * Significant at P<0.05 compared with the control (treated with 0 μM ABA) based on Student’s test.

The transient gene expression system using Arabidopsis mesophyll protoplasts is a sensitive cellular system used to analyze the ABA signal transduction mechanism through ABA-regulated reporter gene constructs [19]. Many important regulatory elements in the 5′ upstream region of gene have been identified as vital motifs required for ABA response [18]. In order to uncover whether the 5′ upstream region of AtVDAC2 contained the motif that suppressed response to ABA, we isolated the 2038bp fragment upstream of the translational start codon of AtVDAC2 coding sequence (pVDAC) using PCR. The pVDAC was then fused to the luciferase gene into the pBI22l-LUC vector in place of CaMV 35S promoter region and the pBI221-pVDAC::LUC vectors was constructed [20] (Figure 2a).

The transient gene expression analysis showed that the pVDAC was also down-regulated by ABA (Figure 2b), which displayed the same tendency as shown in the semi-quantitative RT-PCR test (Figure 1). The promoter activity was inhibited to about 69.8%, 50%, 57% and 27% of the control by 0.1, 1, 10 and 100 μM ABA, respectively (Figure 2). Interestingly, the promoter activity displayed a slight ascending tendency under 10 μM ABA and the similar change tendency could be always gained during our experiments (Figure 2). The probable reason is that there are potential up- or down-regulation motifs in this region, which shows the concentration-dependent effect of ABA on the promoter activity. In our laboratory, an investigation into this potential regulation model is still under way.

### 2.3. Generation of Sense and Antisense AtVDAC2 Transgenic Lines

Earlier studies have demonstrated that transcriptional mechanisms contribute to the regulation of nearly all cellular processes [21]. The time, location, and levels of gene transcripts are finely regulated in organisms, which ensure normal development and increased survival of plant under normal or stressful conditions. Thus, as an important element of mitochondrial in both plant and animal cells, the expression of VDAC affects the functions of mitochondria [22]. Overexpression of a rice VDAC in the Jurkat T-cell line has been shown to be capable of inducing mitochondrial-mediated apoptosis [6,23]. On the other hand, a deficient VDAC may lead to mitochondrial disorders. People with a deficient VDAC present clinically with psychomotor retardation and further biochemical studies on muscle mitochondria have shown an impaired rates of pyruvate oxidation and ATP production, and thus cause the disorder of energy metabolism [24].

**Figure 3.**
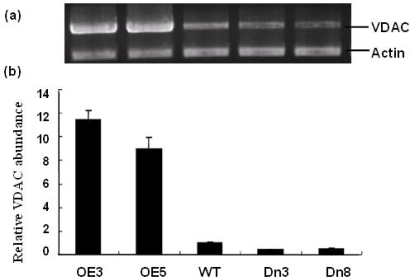
Identification of the AtVDAC2 transgenic Arabidopsis plants by semi-quantitative analysis. (a) The amplified DNA fragment of AtVDAC2 in different transgenic lines were stained by ethidium bromide in agarose gel and the ralative level in each sample was normalized for actin transcripts. (b) The relative amount of AtVDAC2 mRNA were quantified using a software Gel-Pro analyzer 3.0. OE, Dn, WT represent AtVDAC2 sense lines, anti-sense lines and wild type, respectively.

Thus, to further investigate the physiological roles of AtVDAC2 expression pattern responsive to ABA treatment, under- and over-expression of AtVDAC2 mRNA transgenic Arabidopsis plants were obtained by overexpressing sense and antisense AtVDAC2. The positive seedlings on MS with kanamycin were chosen and five independent lines of transgenic plants with sense AtVDAC2 and eight lines with antisense AtVDAC2 were confirmed using the polymerase chain reaction (PCR) analysis. These PCR-positive lines were further analyzed by semi-quantitative analysis and the results showed that the AtVDAC2 mRNA level in sense lines (OE) was about 10 times as that of the control, while in the antisense lines (Dn) the amount of AtVDAC2 mRNA was approximately one-half of the control (Figure 3).

Previous studies derugulated the VDAC expression in cell line or based on mutants could lead to the change of VDAC protein expression level and channel activities such as the cytosolic ATP levels and mitochondrial ATP-synthesis rates. Those results indicate a tight correlation among VDAC gene expression level, protein expression level and channel activity [22,24,25]. As an isoform of VDAC gene family with a high degree of function conservation, AtVDAC2 might be inferred that it has the same change tendency as that of other VDAC isoforms. But whether the derugulation of the AtVDAC2 mRNA can cause the same change tendency at the levels of AtVDAC2 protein expression and channel activities is still needed to be proved by experiments.

### 2.4. The AtVDAC2 Antisense Plants Shown an ABA-Insensitivity Phenotype during the Early Seedling Development in Arabidopsis

ABA has been shown to be capable of arresting restraining the early seedling development, including inhibition of seed germination, blocking the development of green cotyledons and the elongation of the root [15,26]. Accordingly, a comparative analysis in this work was done to investigate whether ABA could affect early seedling growth of the AtVDAC2 transgenic plants.

**Figure 4.**
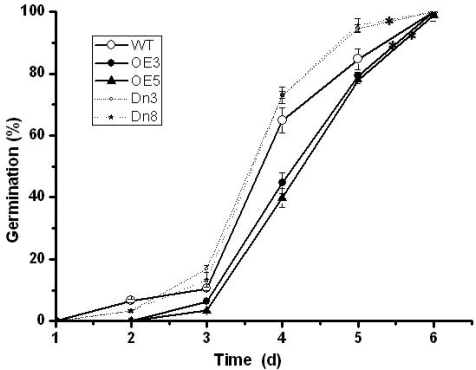
Effect of exogenous ABA on seed germination of different AtVDAC2 tansgenic lines. After stratification, seeds of AtVDAC2 sense (OE) or antisense (Dn) lines and wild-type (WT) were grown with 0.7μM ABA and Germination was scored every 24 hour in two independent seed batches. Values are means ± SD (n=3) from one representative of three independent experiments with similar results. Asterisks indicate significant difference from wild type (P<0.05) based on Student’s test.

Even though there was no visible difference in seeds germination on MS, seeds of wild type and AtVDAC2 overexpression lines germinated more slowly than the AtVDAC2 antisense lines on MS supplemented with 0.7 μM ABA (Figure 4). After four-day imbibitions, maximal differences in germination rate occurred among them. The AtVDAC2 antisense lines had a germination rate about 70 percent, while the germination rate of AtVDAC2 sense lines and wild type seeds was as low as 40 percent and 66 percent, respectively (Figure 4). 100% germination was reached after six days.

**Figure 5.**
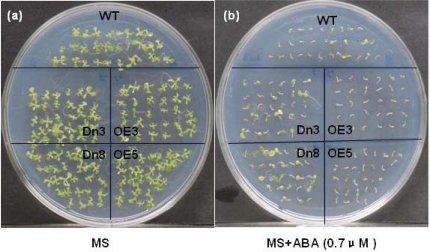
The AtVDAC2 transgenic plants showed different sensitivity to ABA during the early seedling development. Early seedling of AtVDAC2 sense (OE) or antisense (Dn) lines and wild-type (WT) plants after 10 days of growth on control MS (a) or on MS media added with 0.7μM ABA (b). Similar results were obtained in three replicates.

Apart from seed germination, ABA also affected the development of green cotyledons and the elongation of the root of the AtVDAC2 tansgenic lines. After two-week growth on MS media, it was found that there was no visible difference in the radicle emergence and the development of green and expanded cotyledons (Figure 5a). In contrast, in the treatment with 0.7 μM ABA, only the antisense AtVDAC2 lines were found to be able to form the normal green and expanded cotyledons (Figure 5b). Besides, the root length of the antisense AtVDAC2 lines was much longer than the wild type and sense AtVDAC2 plants (Figure 5b). Therefore, the antisense AtVDAC2 lines were shown to be ABA-insensitive phenotypes.

The results presented here show that manipulation of AtVDAC2 alters the plant response to ABA during the early seedling development in Arabidopsis. To gain insights into how AtVDAC2 affects the early seedling development response to ABA, we propose some possible mechanisms of AtVDAC2 involved in ABA signal on the basis of these earlier studies about VDAC isoform. Hexokinase 1 (HXK1) is an interaction protein of VDAC and AtHXK1 over-expression transgenic plants have a faster germination kinetics, which coincides with our observation on the seed germination of AtVDAC2 antisense transgenic lines at the presence of ABA and glucose (Figure 4) [27–29]. On the other hand, the regulation of Ca^2+^ homeostasis by VDAC may involved in the early seedling development [30,31]. Those inferences could provide insights into the function analysis of AtVDAC2 in the further study.

## 3. Experimental Section

### 3.1. Plant Material and Growth Conditions

All Arabidopsis plants in this study were of the ecotype background accessions Reschiev (RLD). Arabidopsis plants were grown on perlite/soil mixture in growth chambers at 23 °C with 16 h light (250 μmol/m ^2^.sec ^1^) and 40% relative humidity.

### 3.2. Construction of Expression Vectors and Isolation of Transgenic Plants

Total RNA was isolated from the leaves of the Arabidopsis plants using the TRIZOL reagent (Invitrogen). After DNase I treatment, the total RNA was reverse transcribed into first stranded cDNA using PrimeScript™ RT reagents Kit (TaKaRa). The open reading frame of AtVDAC2 (At5g67500) was then amplified by PCR from the cDNA with the specific primers, 5′-AACCATGAGCAAAGGTC CAGGACTC-3′ (forward) and 5′-CTCAAGGTTTGAGAGCAAGAGAGAGACC-3′ (reverse). PCR fragments were cloned into pMD18-T vector (Takara) and the resulting plasmid was sequenced. To construct the sense eukaryotic expression vector of AtVDAC2, the vector pMD18-T/AtVDAC2 was digested with Sal I and T4 DNA polymerase followed with an additional digestion with Sac I. The resulting fragment was cloned to the eukaryotic expression vector pCAMBIA2301G which was previously digested with Sma I and Sac I. To obtain antisense eukaryotic expression vector of AtVDAC2, the vector pMD18-T/AtVDAC2 was digested with Sal I and T4 DNA polymerase and then with BamH I. This fragment was cloned to pCAMBIA2301G vector that was previously digested with BamH I and Ecl136 II, After sequencing, these sense and anti-sense transgenic lines were obtained by Agrobacterium-mediated dip flora by using 50mg/L kanamycin as a selective agent [32].

### 3.3. Isolation of Arabidopsis Mesophyll Protoplasts

The isolation of Arabidopsis protoplasts was performed based on a modified protocol [19]. In brief, well-expanded leaves from 3–4 weeks old plants were cut into 0.5–1 mm strips taken from the middle part of the leaves. These strips were then incubated at 23 °C for about 4–5 h with shaking (50 r/min) in an enzyme solution containing 250 mM MES (pH 5.7), 1% cellulase R10, 0.2% macerozyme R10, 0.4 M mannitol, and 20mM KCl. Afterward, washing buffer I containing 167 mM mannitol and 133 mM CaCl_2_ was added to the enzyme solution in an equal-volume and mixed gently. After filtered through a sieve with 150 μm pore diameter, the protoplast suspension was centrifuged at 60 g for 2 min, and the precipitant was then resuspended in washing buffer II containing 333 mM mannitol and 67 mM CaCl_2_. Subsequently, the precipitant was washed twice with Magma solution containing 5 mM MES (pH 5.7), 400 mM mannitol, and 15 mM CaCl_2_. The viability of the protoplasts was verified with fluorescein diacetat staining. The final concentration of protoplast solution was adjusted to 10^6^/mL using Magma solution.

### 3.4. RNA Isolation and Semi-Quantitative RT-PCR

Total RNA was isolated from seedlings and Arabidopsis mesophyll protoplasts using the TRIZOL reagent (Invitrogen). Before RNA isolation, 1-month-old seedlings were pretreated with 30 μM ABA for 0, 2 h, 8 h, 16 h and 24 h and the protoplasts were treated with 0, 5, or 50 μM ABA overnight. To eliminate the contamination of genomic DNA, total RNA was treated with RNase free DNase I. The first-stranded cDNA was synthesized using PrimeScript™ RT reagents Kit (TaKaRa) according to the manufacturer’s instructions. To quantify the amount of VDAC transcripts in each sample, β-actin (AT3G18780) was used as the internal control.

Semi-quantitative RT-PCR was performed using the following primer pairs, 5′-AACCATGAGCA AAGGTCCAGGACTC-3′ (forward) and 5′-CTCAAGGTTTGAGAGCAAGAGAGAGACC-3′ (reverse) for AtVDAC2, 5′-TCCCTCAGCACATTCCAGCAGAT-3′ (forward) and 5′-AACGATTCC TGGACCTGCCTCATC-3′ (reverse) for β-actin. The amplification condition was 94°C (30s), 60°C (30s), and 72°C (30s) for 26 cycles. The PCR products were then separated by agarose gel electrophoresis and quantified by computer analysis of gels stained with ethidium bromide.

### 3.5. Transient Gene Expression in Arabidopsis Protoplasts

The transient gene expression was analyzed in Arabidopsis protoplasts according to a modified protocol [18]. Briefly, for each transfection, 30 μg of pBI221-pVDAC::LUC DNA was added to 200 μL Magma solution containing 5 × 10^5^ protoplasts. While being shaken slowly by hand, PEG solution containing 40% PEG4000, 0.2 M mannitol, and 0.1 M CaCl_2_ in an equal volume was added to the transfection mixture, which was then incubated at room temperature for 10 min to ensure the uptake of the plasmid DNA. After incubation, the transfected protoplasts were washed twice with 1 mL of WI solution containing 4 mM MES (pH 5.7) 500 mM mannitol, and 20 mM KCl, and resuspended in 0.25 mL of WI solution. Finally, the transfected protoplasts were transferred to 24-well tissue culture plates (0.25 mL in each well) and incubated in the dark at 23 °C overnight.

For ABA treatment, the transfected protoplasts were treated with ABA for another 16 h at different concentrations of 0, 0.1, 1, 10, or 100 μM.

The luciferase activity of the treated protoplasts were measured using Luciferase detection kit (Promega) according to the manufacturer’s instructions. The actual value was normalized by protein content of each sample and expressed as the relative luminescence units (RLU)/mg of protein.

### 3.6. Phenotype Analysis of the AT VDAC2 Transgenic Plants

Seeds of transgenic and wild-type plants were harvested and stored at room temperature for at least four weeks. After storage, seeds were surface sterilized with 0.1% mercuric chloride and sown on Murashige and Skoog (MS) agar plates supplemented with 0.7 μM ABA. After stratification at 4 °C in the dark for three days, the plates were transferred into growth chambers. The characteristics of the early seedling development were observed every 24 h, including germination, the development of green cotyledons, and the elongation of the root. The experiments were repeated for three times.

## 4. Conclusions

In this paper, we investigated the expression pattern of AtVDAC2 in the response to ABA in *A. thaliana* using RT-PCR and the protoplast transient expression system. The results showed that ABA suppressed the accumulation of AtVDAC2 transcripts. Further phenotype analysis of the stable AtVDAC2 transgenic plants confirmed that AtVDAC2 involved in ABA signaling during the seed germination and the development of green cotyledons. Our study could provide insights into the function analysis of AtVDAC2. Much work still needs to be done for the molecular elucidation of AtVDAC2 expression characteristics and its role in ABA signaling in *A. thaliana*.
